# ThPOK Inhibits Osteoclast Formation Via NFATc1 Transcription and Function

**DOI:** 10.1002/jbm4.10613

**Published:** 2022-03-14

**Authors:** Wei Zou, Takashi Izawa, Nidhi Rohatgi, Steven Y Zou, Yongjia Li, Steven L Teitelbaum

**Affiliations:** ^1^ Division of Anatomic and Molecular Pathology, Department of Pathology and Immunology Washington University School of Medicine St. Louis MO USA; ^2^ Department of Orthodontics Graduate School of Medicine, Dentistry and Pharmaceutical Sciences, Okayama University Okayama Japan; ^3^ Department of Pharmacology Jiangsu University School of Medicine Zhenjiang China; ^4^ Division of Bone and Mineral Diseases, Department of Medicine Washington University School of Medicine St. Louis MO USA

**Keywords:** OSTEOCLASTS, CELLS OF BONE, CELL/TISSUE SIGNALING, TRANSCRIPTION FACTORS, OSTEOPOROSIS, DISEASES AND DISORDERS OF/RELATED TO BONE

## Abstract

Both LRF (*Zbtb7a*) and ThPOK (*Zbtb7b*) belong to the POK (BTB/POZ and Kruppel) family of transcription repressors that participate in development, differentiation, and oncogenesis. Although LRF mediates osteoclast differentiation by regulating NFATc1 expression, the principal established function of ThPOK is transcriptional control of T‐cell lineage commitment. Whether ThPOK affects osteoclast formation or function is not known. We find that marrow macrophage ThPOK expression diminishes with exposure to receptor activator of NF‐kB ligand (RANKL), but ThPOK deficiency does not affect osteoclast differentiation. On the other hand, enhanced ThPOK, in macrophages, substantially impairs osteoclastogenesis. Excess ThPOK binds the NFATc1 promoter and suppresses its transcription, suggesting a mechanism for its osteoclast inhibitory effect. Despite suppression of osteoclastogenesis by excess ThPOK being associated with diminished NFATc1, osteoclast formation is not rescued by NFATc1 overexpression. Thus, ThPOK appears to inhibit NFATc1 transcription and its osteoclastogenic capacity, while its depletion has no effect on the bone‐resorptive cell. © 2022 The Authors. *JBMR Plus* published by Wiley Periodicals LLC on behalf of American Society for Bone and Mineral Research.

## Introduction

Osteoclasts, the principal if not exclusive bone‐resorbing cells, differentiate from macrophage precursors after stimulation with receptor activator of NF‐κB ligand (RANKL) and macrophage colony‐stimulating factor (M‐CSF). Although M‐CSF is crucial for survival and proliferation of osteoclast precursors,^(^ ^1^, ^2^ ^)^ RANKL is the key cytokine inducing osteoclast differentiation. Binding of RANKL to its receptor, receptor activator of NF‐κB (RANK), recruits the adaptor molecule TRAF6 to this complex,^(^ ^3^ ^)^ which, in turn, activates the NF‐κB, Akt, and MAPK pathways, the latter including c‐jun N‐terminal kinase (JNK) and p38.^(^ ^4^, ^5^, ^6^ ^)^ These immediate signals also stimulate the activator protein‐1 (AP‐1) transcription factor complex by inducing c‐Fos expression.^(^ ^7^ ^)^ Activated NF‐κB and AP1 target the NFATc1 promoter to induce osteoclastogenesis.^(^ ^8^, ^9^ ^)^ The importance of NFATc1 in osteoclast development is confirmed by its overexpression prompting embryonic stem cells to differentiate into the bone‐resorbing polykaryons in a RANKL‐independent manner.^(^ ^8^ ^)^

Poxvirus and zinc finger (POZ) and Kruppel‐type (POK) proteins are members of a family of transcription factors that participate in development and differentiation.^(^ ^10^, ^11^, ^12^ ^)^ Dysregulated expression of POK family members is also associated with various cancers. ThPOK, like other POK proteins, has N‐terminal BTB/POZ and C‐terminal zinc finger domains, each of which participate in cell differentiation and oncogenesis.^(^ ^11^, ^13^ ^)^ ThPOK is conserved in vertebrates, implying evolutionarily significant function.^(^ ^11^ ^)^ Currently, the most prominent feature of ThPOK is its transcriptional control of CD4/CD8 T‐cell lineage commitment by repressing genes involved in CD8 differentiation and activating those modulating CD4 cell differentiation.

BCL6, a POK family member, suppresses expression of osteoclast differentiation genes, thus negatively regulating the cell's maturation.^(^ ^14^ ^)^ LRF/Pokemon is a relative of ThPOK in vertebrates and mediates osteoclast differentiation by regulating NFATc1 transcription.^(^ ^15^ ^)^ These activities by its family members raised the possibility that ThPOK may also influence bone resorption. In fact, ThPOK collaborates with NF‐κB by delivering it to its DNA binding elements, thus promoting interchromosomal interactions.^(^ ^16^ ^)^ We find, however, that while osteoclast differentiation of cultured ThPOK‐deficient bone marrow macrophages (BMMs) mirrors that of wild‐type (WT) control, overexpressed ThPOK regulates NFATc1 transcription and inhibits its function, substantially impairing osteoclastogenesis even in the presence of normal NFATc1 expression. Thus, excess ThPOK, as occurs in various cancers,^(^ ^17^ ^)^ may suppress osteoclastogenesis, thus protecting the skeleton.

## Materials and Methods

### Mice

C57BL/6J WT mice (cat. #000664) were purchased from Jackson Laboratory (Bar Harbor, ME, USA), and all mice were 8 to 10 weeks old at time of study. They were housed in the animal care unit of Washington University School of Medicine, where they were maintained according to guidelines of the Association for Assessment and Accreditation of Laboratory Animal Care. ThPOK^−/−^ mice were kindly provided by Dr Takeshi Egawa (Washington University School of Medicine). All animal experimentation was approved by the Animal Studies Committee of Washington University School of Medicine.

### Reagents

Recombinant murine M‐CSF was obtained from R&D Systems (Minneapolis, MN, USA). Glutathione *S*‐transferase (GST)‐RANKL was expressed in our laboratory as described.^(^ ^18^ ^)^ The source of antibodies is as follows: mouse anti‐Flag and anti‐actin monoclonal antibodies from Sigma (St. Louis, MO, USA); mAb 327, directed against the c‐Src protein, were gifts of Dr A Shaw (Department of Pathology, Washington University School of Medicine, St. Louis, MO, USA); anti‐pERK, pAKT, pJNK, pP38, pIkBa antibodies and anti β3 integrin, ThPOK, ERK, AKT, JNK, and P38 antibodies were purchased from Cell Signaling (Beverly, MA, USA); anti‐RANK (IMG‐325A) antibody from Imgenex (Novus Biologicals, Centennial, CO, USA); and anti‐NFATc1 and anti‐c‐Fms antibodies from Santa Cruz Biotechnology Inc (Dallas, TX, USA). All other chemicals were obtained from Sigma.

### Macrophage isolation and OC culture

Primary BMMs were prepared as described previously with slight modification. Marrow was extracted from femora and tibias of 8‐ to 10‐week‐old mice with α‐MEM and cultured in α‐MEM containing 10% inactivated fetal bovine serum, 100 IU/mL penicillin, and 100 μg/mL streptomycin (α‐10 medium) with 1:10 CMG condition media on petri plastic dishes. Cells were incubated at 37°C in 5% CO_2_ for 3 days and then washed with PBS and lifted with 1× trypsin/EDTA (Invitrogen, Carlsbad, CA, USA) in PBS. A total of 1.2 × 10^4^ cells were cultured in 500 μL α‐MEM containing 10% heat‐inactivated FBS with 100 ng/mL GST‐RANKL and 30 ng/mL of mouse recombinant M‐CSF in 48‐well tissue culture plates, some containing sterile bone slices. Cells were fixed and stained for tartrate‐resistant acid phosphatase (TRAP) activity after 5 days in culture, using a commercial kit (Sigma 387‐A).

### Actin ring staining

For actin ring staining, cells were cultured on bovine bone slice in the presence of M‐CSF and RANKL for 6 days, at which time cells were fixed in 4% paraformaldehyde, permeabilized in 0.1% Triton X‐100, rinsed in PBS, and immunostained with Alexa 543 phalloidin (Molecular Probes, Eugene, OR, USA).

### Actin ring staining and bone resorption assay

We generated osteoclasts on bone slices from bone marrow‐derived macrophages by exposure to 100 ng/mL RANKL and 30 ng/mL M‐CSF. For actin ring staining, the cells were fixed in 4% paraformaldehyde and permeabilized in 0.1% Triton X‐100, rinsed in PBS, and immunostained with AlexaFluor 488‐phalloidin (Invitrogen). For bone resorption assay, osteoclasts were removed and resorption pits were visualized by incubation of the specimen with 20 μg/mL peroxidase‐conjugated wheat germ agglutinin (Sigma) for 1 hour and stained with 3,3′‐diaminobenzidine (Sigma).

### Plasmids and retroviral transduction

Wild‐type mouse ThPOK cDNA was subcloned into the BamH1 and Xho1 sites of a pMX Flag tagged retroviral vector. R389G mutant was generated using the QuickChange Site‐Directed Mutagenesis Kit (Stratagene, La Jolla, CA, USA). WT and R389G mutant ThPOK cDNA were transfected transiently into Plat‐E packaging cells using calcium phosphate. The medium was changed on the next day, and virus was collected 48 hours after transfection. BMMs were infected with virus for 24 hours in the presence of 1:10 CMG and 4 μg/mL polybrene (Sigma). Cells were selected in the presence of M‐CSF and 2 μg/mL puromycin (Calbiochem) for 3 days before use as OC precursors.

### Western blotting and immunoprecipitation

Cultured cells were washed twice with ice‐cold PBS and lysed in RIPA buffer containing 20 mM Tris, pH 7.5, 150 mM NaCl, 1 mM EDTA, 1 mM EGTA, 1% Triton X‐100, 2.5 mM sodium pyrophosphate, 1 mM β‐glycerophosphate, 1 mM Na3VO4, 1 mM NaF, and 1× protease inhibitor mixture (Roche, Mannheim, Germany). After incubation on ice for 10 minutes, cell lysates were clarified by centrifugation at 21,000g for 10 minutes. Forty micrograms of total lysates were subjected to 8% sodium dodecyl sulfate polyacrylamide gel electrophoresis and transferred onto PVDF membranes. Filters were blocked in 0.1% casein in PBS for 1 hour and incubated with primary antibodies at 4°C overnight followed by probing with fluorescence‐labeled secondary antibodies (Jackson Laboratory). Proteins were detected with the Odyssey Infrared Imaging System (LI‐COR Biosciences, Lincoln, NE, USA).

### RNA extraction and quantitative qPCR

RNA from cultured cells was isolated and purified using the RNeasy RNA purification kit (Qiagen, Valencia, CA, USA); RLT lysis buffer was supplemented with β‐mercaptoethanol (1%). cDNA was synthesized from RNA (1 μg) using the High Capacity cDNA Reverse Transcription kit (Applied Biosystems, Carlsbad, CA, USA). Real‐time PCR was performed using the SYBR Green Master Mix Kit and gene‐specific primers. The quantitative PCR reaction was performed on ABI PRISM 7500 sequence detection system (Applied Biosystems). All reactions were performed in triplicate and relative mRNA levels were calculated by the comparative threshold cycle method using GAPDH as an internal control. The sequence of the oligonucleotides used in quantitative real‐time‐PCR analyses were as follows: ThPOK (*Zbtb7b*): Forward 5′‐CCCGAGGATGACCTGATTGG‐3′; reverse: 5′‐CCTGCGTCCTGATGGTGAG‐3′; LRF (*Zbtb7a*): forward 5′‐CTTTGCGACGTGGTGATTCTT‐3′; reverse 5′‐CGTTCTGCTGGTCCACTACA‐3′; *Itgb3*; forward 5′‐CCACACGAGGCGTGAACTC‐3′; reverse 5′‐CTTCAGGTTACATCGGGGTGA‐3′; *TRAP*: forward 5′‐CACTCCCACCCTGAGATTTGT‐3′; reverse 5′‐CATCGTCTGCACGGTTCTG‐3′; *Cathepsin K*: forward 5′‐GAAGAAGACTCACCAGAAGCAG‐3′; reverse 5′‐TCCAGGTTATGGGCAGAGATT‐3′; *OC‐Stamp*: forward 5′‐CTGTAACGAACTACTGACCCAGC‐3′; reverse: 5′‐CCCAGGCTTAGGAAGACGAAG‐3′; *DC‐Stamp*: forward 5′‐GGGGACTTATGTGTTTCCACG‐3′; reverse 5′‐ACAAAGCAACAGACTCCCAAAT‐3′; *Gapdh*: forward 5′‐AGGTCGGTGTGAACGGATTTG‐3′; reverse 5′‐TGTAGACCATGTAGTTGAGGTCA‐3′.

### Chromatin immunoprecipitation (ChIP) assay

For immunoprecipitation, 30 μL of magnetic protein A beads were used. The beads were washed twice with PBS containing 0.02% tween 20. After the final wash, beads were resuspended with the antibody overnight at 4°C. BMMs (5 × 10^6^) transduced with vector or ThPOK were plated in 150 mm tissue culture plates in the presence of M‐CSF and RANKL (100 ng/mL) for 2 days. Formaldehyde was added directly to cell culture media for 10 minutes at room temperature such that the final concentration was 1%. Cross‐linking was quenched by the addition of glycine to a final concentration of 0.125 M. Cells were washed three times with PBS, scraped off the plates in a small amount of PBS, and centrifuged, and the pellet was washed with PBS containing protease inhibitors (Complete, Roche). Cells were then resuspended in 1 mL PBS, and protease inhibitors centrifuged at 2,400g for 5 minutes at 4°C. The pellet was resuspended in cold sonication buffer (50 mM tris(hydroxymethyl)aminomethane [Tris]–HCl pH 8, 10 mM EDTA, 0.1% sodium dodecyl sulfate [SDS], 0.5% sodium deoxycholate and protease inhibitors) for 25 to 30 minutes on ice. Cells were chromatin sheared using Bioruptor (6 cycles, 10 minutes at high speed per cycle). The samples were centrifuged to pellet the cellular debris. Five percent of the cells were collected in a separate tube to be used as input control. The supernatant was divided equally between immunoprecipitation samples to include isotype control. Sheared DNA was incubated with the bead‐antibody slurry. The next day, DNA‐protein complexes were washed in low‐salt buffer (SDS 0.1%, Triton X‐100 1%, EDTA 2 mM, Tris‐HCl pH 8.0 20 mM, NaCl 150 mM) followed by high‐salt buffer (SDS 0.1%, Triton X‐100 1%, EDTA 2 mM, Tris‐HCl pH 8.0 20 mM, NaCl 500 mM), LiCl buffer (LiCl 0.25 M, nonidet P‐40 1%, deoxycholate 1%, EDTA 1 mM, Tris‐HCl pH 8.0, 10 mM), and Tris‐EDTA buffer. DNA was eluted by adding 250 μL of elution buffer (SDS 1%, NaHCO_3_ 0.1 M). Samples and inputs were de‐cross‐linked and cleaned for quantitative polymerase chain reaction (qPCR) analysis. We used antibodies against Flag (Clone M2, Sigma) and normal mouse IgG (Jackson ImmunoResearch, West Grove, PA, USA). The sequence of NFATc1 primers were: 5′‐AAGCGCTTTTCCAAATTTCC‐3′ (sense) and 5′‐CCTGAGAAAGCTACTCTCCCTTT‐3′ (antisense).

### Statistical analysis

Data are expressed as mean ± SD. Statistical analyses were performed with Prism software version 8 (GraphPad Software, La Jolla, CA, USA) using unpaired Student's 2‐tailed *t* test, one‐way or two‐way ANOVA test with Holm‐Sidak post hoc test with adjustment for multiple testing. In all experiments, statistical significance was considered when *p < 0.05, **p < 0.01, ***p < 0.001 in all experiments.

## Results

### ThPOK deficiency does not affect osteoclastogenesis

**Fig. 1:**
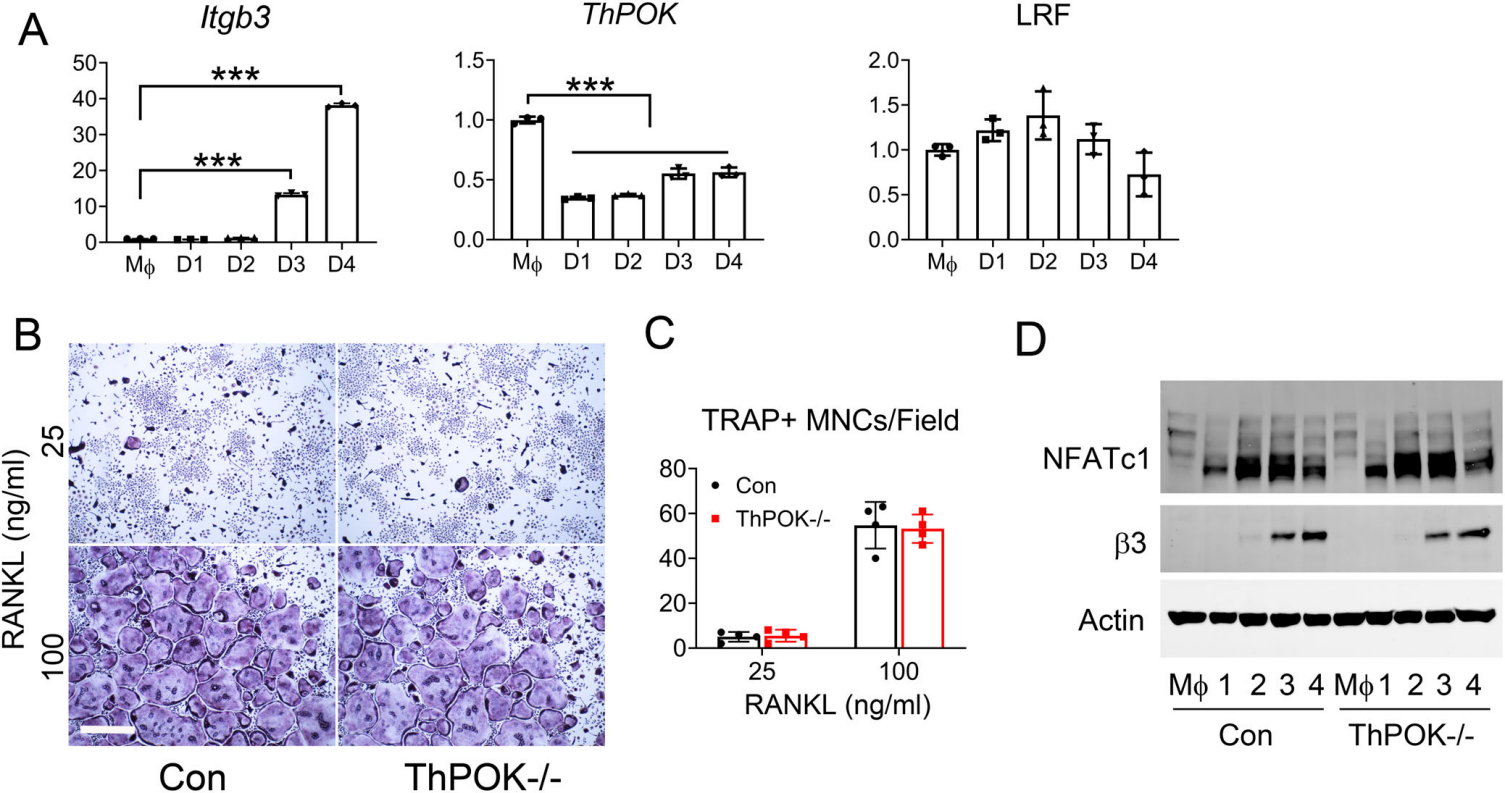
Deficiency of ThPOK does not affect osteoclast differentiation. (*A*) WT BMMs were exposed to M‐CSF and RANKL (100 ng/mL) with time. β3 integrin and ThPOK, LRF mRNA expression was determined by qPCR. (*B*) Con or ThPOK^−/−^ macrophages were cultured with M‐CSF and RANKL for 5 days on tissue culture plates and TRAP stained. Scale bar = 500 μm. (*C*) Number of TRAP*+* osteoclasts/field illustrated in *B*. (*D*) Con or ThPOK^−/−^ macrophages were cultured with RANKL and M‐CSF for indicated days. Cells cultured with M‐CSF for 4 days serve as con (Mϕ). Osteoclast differentiation markers NFATc1 and β3 were detected by immunoblot. Actin serves as loading control. Data are expressed as mean ± SD. Statistical analyses were performed with one‐way (*A*) ANOVA test with Holm–Sidak post hoc test with adjustment for multiple testing. ***p < 0.001.

To determine the expression pattern of ThPOK proteins during osteoclast differentiation, BMMs isolated from WT mice were exposed to RANKL and M‐CSF. Consistent with osteoclastogenesis, β3 integrin mRNA increases (Fig. 1*A*). In contrast, ThPOK mRNA is abundant in BMMs but diminishes with generation of the resorptive cell. Although not significant, LRF expression tends to increase with early RANKL stimulation (Fig. 1*A*).

Because it is highly expressed in macrophages but is significantly decreased after addition of RANKL, we asked if ThPOK participates in osteoclast differentiation. Hence, we cultured WT and ThPOK^−/−^ BMMs in M‐CSF and various amounts of RANKL for 5 days, and determined TRAP activity. Surprisingly, the abundance and appearance of osteoclasts generated in vitro, from ThPOK−/− BMMS, using various amounts of RANKL, are indistinguishable from their WT counterparts (Fig. 1*B*, *C*). This conclusion is in keeping with parallel increases in NFATc1 and β3 integrin subunit expression as WT and ThPOK^−/−^ cells differentiate (Fig. 1*D*).

### Overexpression of ThPOK in macrophages inhibits osteoclastogenesis

**Fig. 2:**
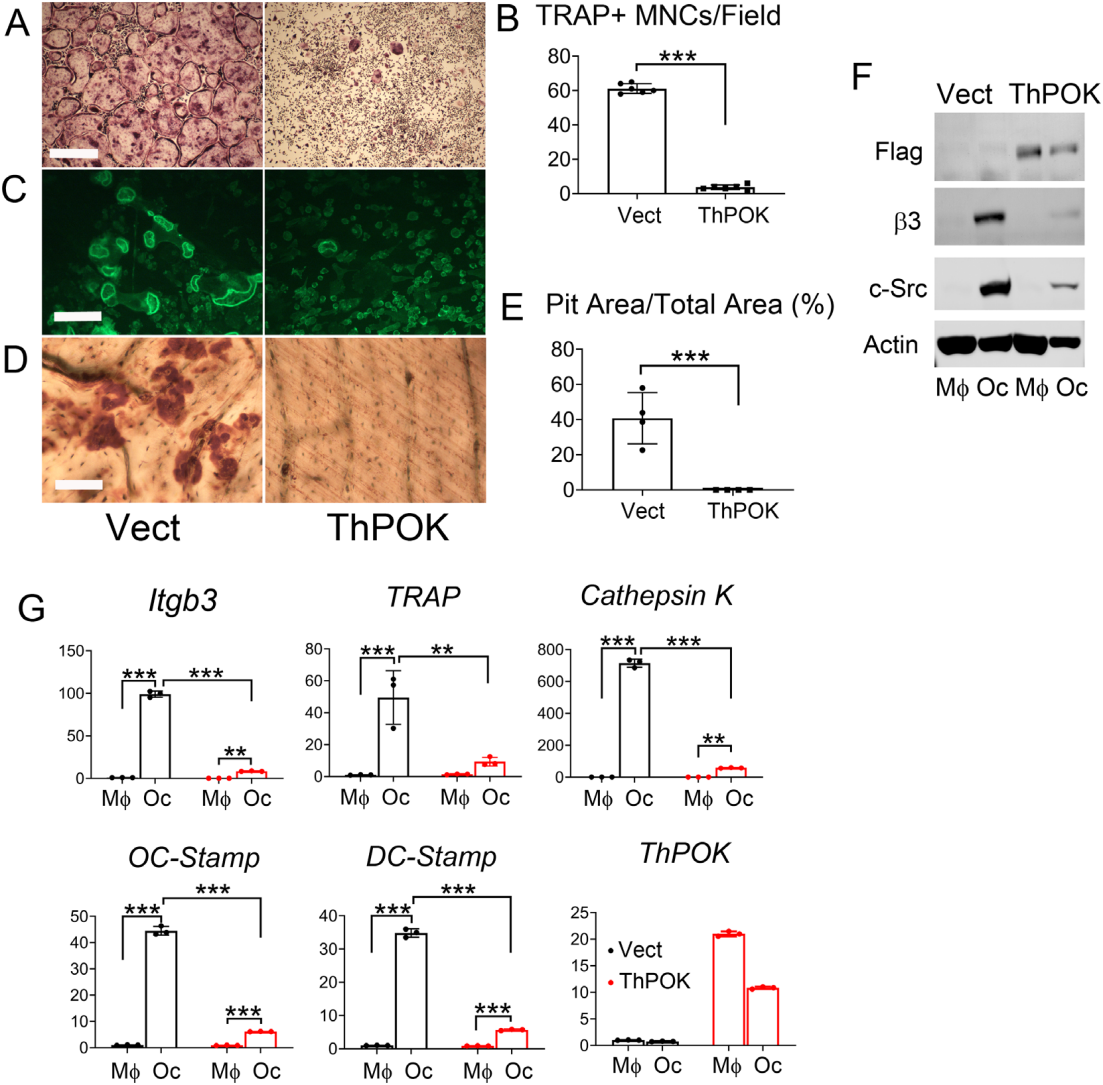
Excess ThPOK inhibits osteoclast differentiation. (*A–E*) BMMs retrovirally transduced with ThPOK or vector were cultured on plates with M‐CSF and RANKL for 5 days (*A*) or for 6 days on bone slices (*C*, *D*). Cells were stained for TRAP activity (*A*), phalloidin to identify actin rings (*C*), or cells were removed and the bone slices stained with peroxidase‐labeled (*D*) wheat germ agglutinin to visualize resorption lacunae. (*B*) Number of TRAP^+^ osteoclasts/field illustrated in *A*. (*E*) Quantification analysis of pit area in *D*. (*F*, *G*) BMMs retrovirally transduced with ThPOK (Flag‐tagged ThPOK) or vector were cultured with M‐CSF and RANKL (OC) or only M‐CSF (Mϕ) for 5 days. Osteoclast differentiation markers were determined by immunoblot (*F*) or qPCR (*G*). Scale bar = 500 μm (*A*) and 100 μm (*C*, *D*). Data are expressed as mean ± SD. Statistical analyses were performed with unpaired *t* test (*B*, *E*) or two‐way (*G*) ANOVA test with Holm–Sidak post hoc test with adjustment for multiple testing. *p < 0.05, **p < 0.01, ***p < 0.001.

Despite failure of ThPOK deletion to influence formation of the resorptive cell, its decreased expression with osteoclast differentiation raised the possibility that abundant ThPOK may inhibit the process. To determine if such is the case, we retrovirally transduced WT ThPOK or vector into WT BMMs. The transduced cells were exposed to M‐CSF and RANKL for 5 days. TRAP staining revealed ThPOK overexpression decreases osteoclast formation (Fig. 2*A*, *B*). Additionally, actin rings (Fig. 2*C*), key organelles of the resorptive process, and resorptive lacunae (Fig. 2*D*, *E*) were virtually eliminated in ThPOK‐overexpressing cells cultured on bone. Confirming these morphological abnormalities reflect impaired osteoclast differentiation, the β3 integrin subunit and c‐Src, both characteristic of the mature resorptive cell, were diminished in ThPOK‐expressing osteoclasts (Fig. 2*F*). The same holds true regarding mRNA abundance of β3 integrin, TRAP, cathepsin K, OC‐Stamp, and DC‐Stamp (Fig. 2*G*). Thus, although absence of the transcription factor has no effect on osteoclastogenesis, enhanced expression of ThPOK, as occurs in lymphoma,^(^ ^17^ ^)^ suppresses osteoclast formation.

### ThPOK expression does not change RANK and c‐Fms expression by BMMs

**Fig. 3:**
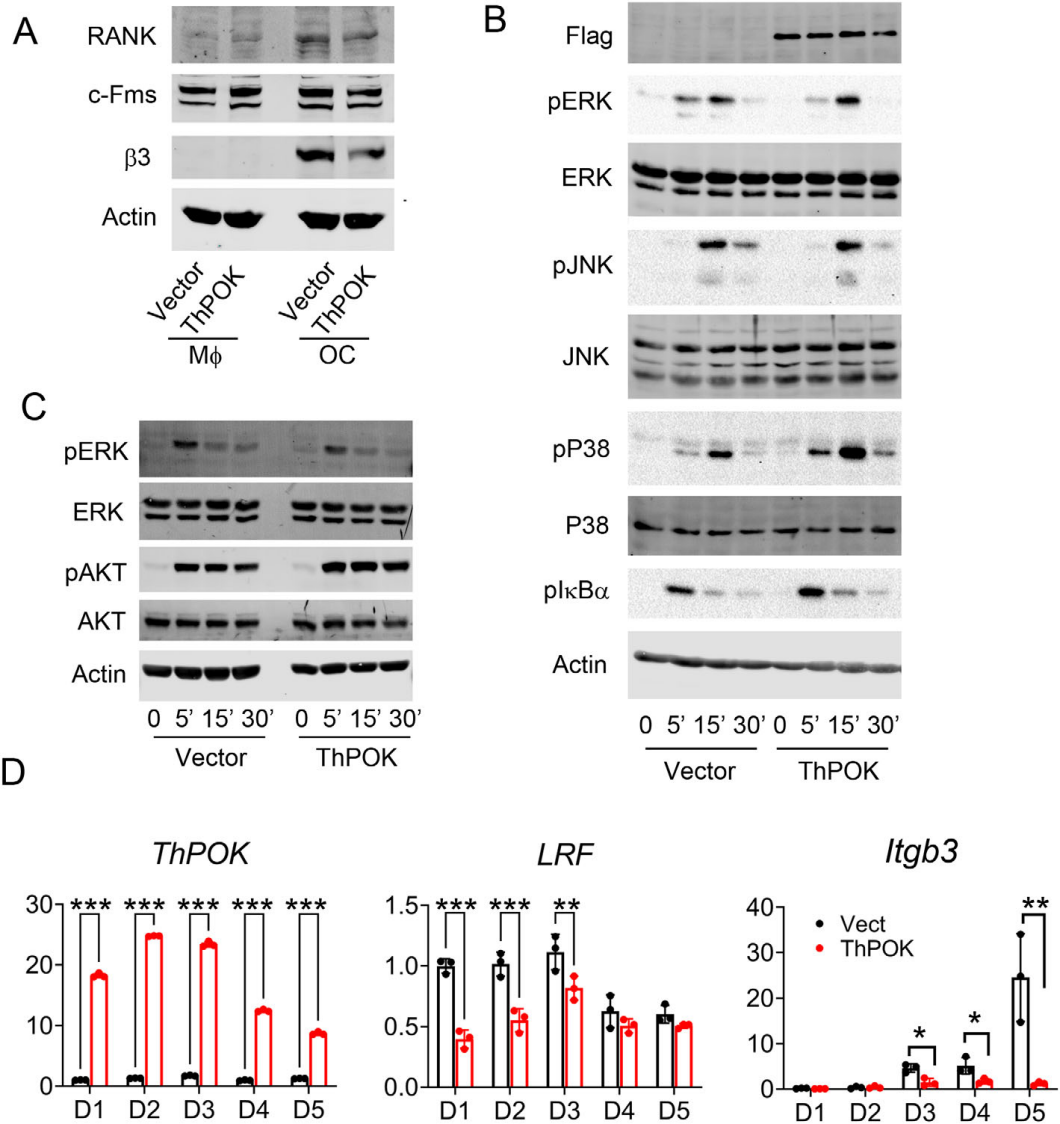
ThPOK does not change RANK and c‐Fms expression. (*A*) BMMs retrovirally transduced with ThPOK or vector were cultured with M‐CSF alone (Mϕ) or M‐CSF and RANKL (OC) for 3 days. RANK, c‐Fms, and β3 integrin expression were determined by immunoblotting. Actin serves as loading control. (*B*, *C*) Vector or Flag‐tagged ThPOK transduced WT BMMs were serum‐ and cytokine‐starved overnight. The cells were then exposed to either 100 ng/mL RANKL (*B*) or 100 ng/mL M‐CSF (*C*) with time. Signaling molecules were identified by immunoblotting. Actin serves as a loading control. (*D*) BMMs retrovirally transduced with ThPOK or vector were exposed to M‐CSF and RANKL (100 ng/mL) with time. ThPOK, LRF, and β3 integrin mRNA expression was determined by qPCR. Data are expressed as mean ± SD. Statistical analyses were performed with two‐way ANOVA test with Holm–Sidak post hoc test with adjustment for multiple testing. *p < 0.05, **p < 0.01, ***p < 0.001.

The suppressive effects of ThPOK on osteoclastogenesis raised the possibility that the transcription factor reduces abundance of M‐CSF receptor (c‐Fms) or RANK on progenitors. Retrovirally transduced WT ThPOK or vector into WT BMMs, however, alters neither RANK nor c‐Fms abundance (Fig. 3*A*). Additionally, ThPOK overexpression does not impact RANKL‐induced osteoclastogenic signaling, including NF‐κB activation, manifest by IκB phosphorylation, nor c‐Jun N‐terminal kinase, ERK1/2, and p‐38 phosphorylation (Fig. 3*B*). M‐CSF‐stimulated ERK1/2 and AKT phosphorylation are also normal in ThPOK overexpressing macrophages (Fig. 3*C*). ThPOK, therefore, appears to suppress osteoclast formation independent of the two key inductive cytokines.

Ectopic expression of LRF in WT BMMs completely inhibits their differentiation into osteoclasts. Because they belong to the same subgroup of POK factors, we asked if ThPOK inhibition of osteoclastogenesis is due to upregulation of LRF expression. Thus, we transduced WT BMMs with ThPOK and cultured them with RANKL and M‐CSF. ThPOK and LRF expression were analyzed with time. Although overexpression of ThPOK dramatically reduces the osteoclast differentiation marker integrin β3, it decreases LRF expression at the early stage of osteoclastogenesis (day 1 to day 3). Therefore, ThPOK inhibition of osteoclastogenesis is not mediated by enhanced LRF (Fig. 3*D*).

### ThPOK inhibits NFATc1 expression

**Fig. 4:**
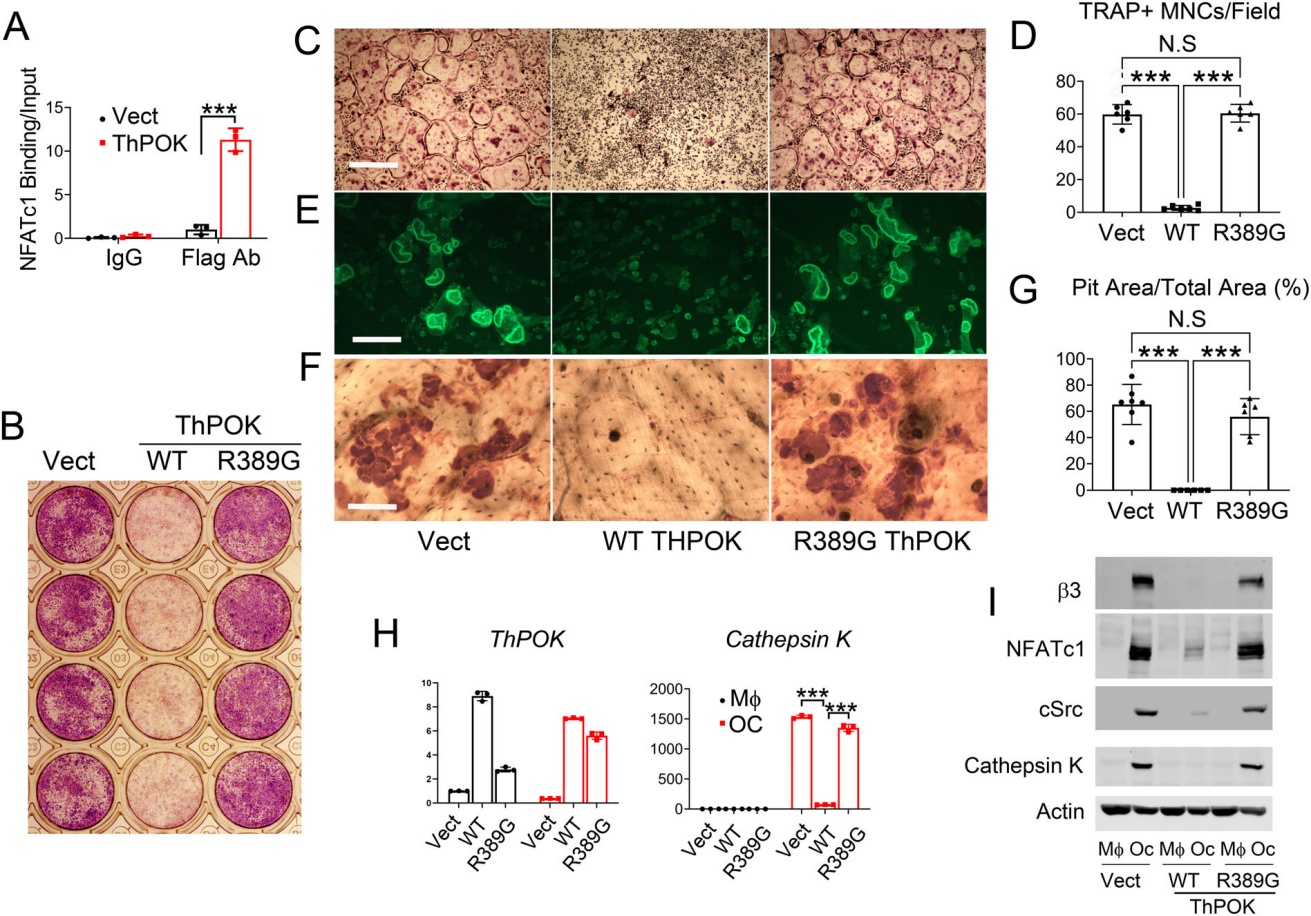
ThPOK inhibits NFATc1 expression. (*A*) Vector or Flag‐tagged ThPOK transduced BMMs were exposed to M‐CSF and RANKL (100 ng/mL) for 2 days. ThPOK (Flag) binding to NFATc1 response element in the NFATc1 promoter was determined by ChIP assay. Immunoglobulin G (IgG) and vector transduced cells serve as control. (*B–G*) BMMs retrovirally transduced with vector, WT, or R389G ThPOK were cultured on plates with M‐CSF and RANKL for 5 days (*B*, *C*) or for 6 days on bone slices (*E*, *F*). Cells were stained for TRAP activity (*B*, *C*). Phalloidin to identify actin rings (*E*) or cells were removed and the bone slices stained with peroxidase‐labeled wheat germ agglutinin to visualize resorption lacunae (*F*). (*D*) Number of TRAP^+^ osteoclasts/field illustrated in *C*. (*G*) Quantification analysis of pit area in *F*. (*H*, *I*) BMMs retrovirally transduced with vector, WT, or R389G ThPOK were cultured with M‐CSF and RANKL (OC) or only M‐CSF (Mϕ) for 5 days. Osteoclast differentiation markers were determined by qPCR (*H*) or immunoblot (*I*). Scale bar = 500 μm (*C*) and 100 μm (*E*, *F*). Data are expressed as mean ± SD. Statistical analyses were performed with one‐way ANOVA (*D*, *G*) or two‐way (*A*, *H*) ANOVA test with Holm–Sidak post hoc test with adjustment for multiple testing. ***p < 0.001.

LRF (*Zbtb7a*) negatively regulates osteoclast differentiation by blunting NFATc1 transcription.^(^ ^15^ ^)^ Given their relationship, we asked if ThPOK may do the same. Indicating such may be the case, ThPOK binds the proximal NFATc1 promoter (Fig. 4*A*).

Arginine at residue 389 (R389), located within the second zinc finger domain, is essential for ThPOK's DNA binding activity.^(^ ^19^ ^)^ Confirming that ThPOK transcriptionally suppresses osteoclastogenesis, its inactivating mutant, ThPOK^R389G^, obviates its inhibitory effect on osteoclast differentiation, bone resorption (Fig. 4*B–H*), and NFATc1 expression (Fig. 4*I*).

### ThPOK inhibits NFATc1 function

**Fig. 5:**
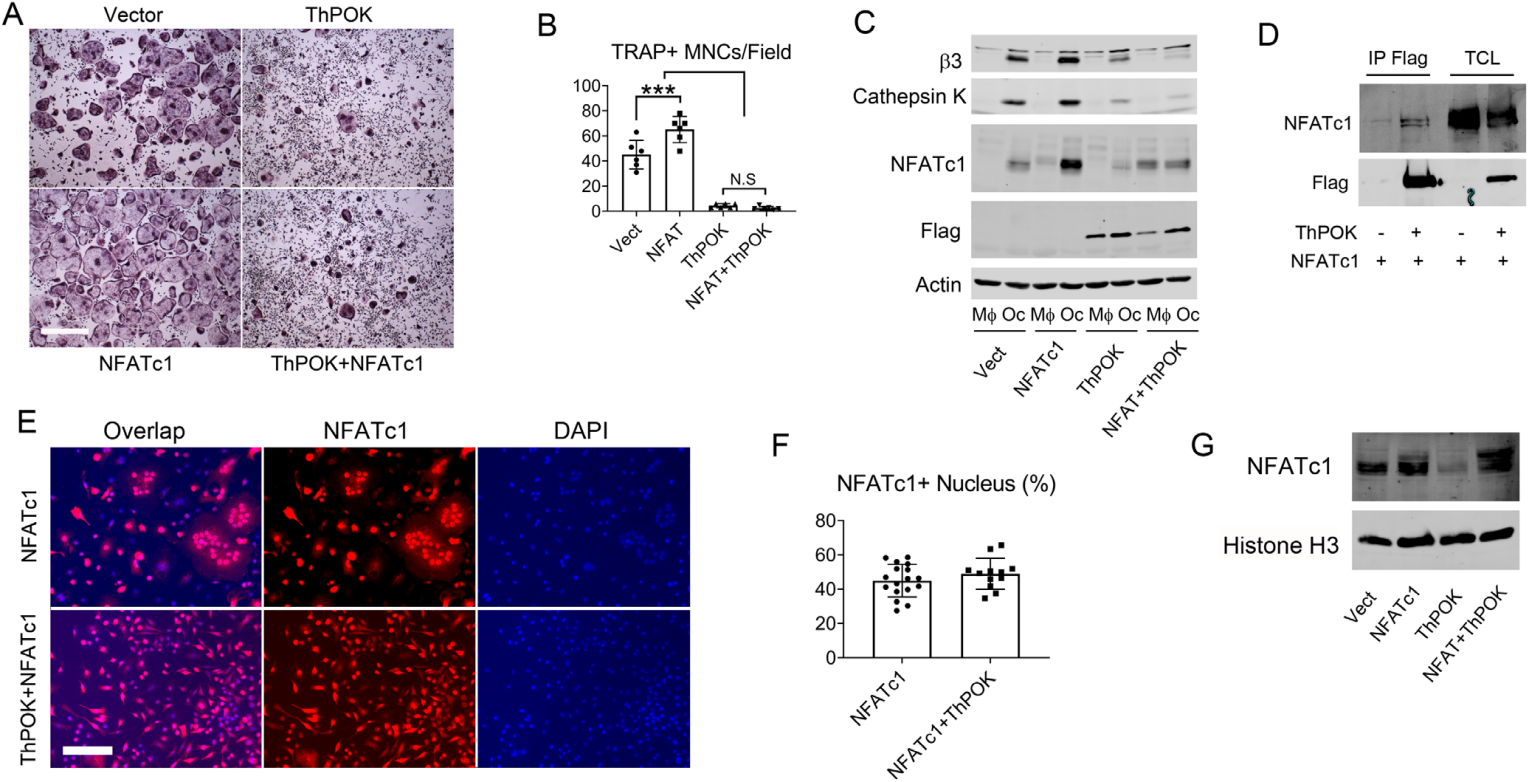
ThPOK inhibits NFATc1 function. (*A–C*) WT BMMs were retrovirally transduced with ThPOK and/or NFATc1. Vector transduced BMMs serve as control. Cells were cultured with M‐CSF and RANKL (50 ng/mL) for 5 days after which they were (*A*) TRAP stained and (*C*) assayed for osteoclast differentiation markers by immunoblot. Scale bar = 500 μm. (*B*) Number of TRAP^+^ osteoclasts/field illustrated in *A*. (*D*) A total of 293 T cells were transfected with Flag‐tagged ThPOK and NFATc1. Flag (ThPOK) immunoprecipitates were immunoblotted for NFATc1 and FLAG. (*E–G*) WT BMMs were retrovirally transduced with NFATc1 alone or NFATc1 with ThPOK. Cells were cultured with M‐CSF and RANKL (50 ng/mL) for 4 days after which (*E*) cells were stained with NFATc1 by IF or (*G*) NFATc1 expression in nuclear lysis was determined by immunoblot. Histone H3 as loading control. Scale bar = 100 μm. (*F*) Number of NFATc1^+^ nucleus illustrated in *E*. Data are expressed as mean ± SD. Statistical analyses were performed with one‐way ANOVA (*B*) with Holm–Sidak post hoc test with adjustment for multiple testing or unpaired *t* test (*F*). ***p < 0.001.

Abundant NFATc1 can induce osteoclast formation even in the absence of RANKL.^(^ ^8^ ^)^ To determine if NFATc1 overexpression prevents ThPOK's inhibitory effect on osteoclastogenesis, WT BMMs were retrovirally transduced with ThPOK and/or NFATc1. Vector‐transduced BMMs serve as control. NFATc1 overexpression and its stimulation of osteoclastogenesis were confirmed by TRAP staining and Western blot (Fig. 5*A–C*). Co‐IP indicates ThPOK and NFATc1 form a complex (Fig. 5*D*) and ThPOK completely blocked osteoclast differentiation even in NFATc1‐expressing cells (Fig. 5*A–C*). To determine if ThPOK affects NFATc1 nuclear translocation, we quantified the transcription factor, in nuclei, by IF or immunoblot in osteoclasts transduced with NFATc1 and/or ThPOK. As shown in Fig. 5*E–G*, presence of ThPOK does not affect NFATc1 nuclear translocation in osteoclasts. Thus, ThPOK appears to suppress osteoclast formation by transcriptionally blunting NFATc1 expression and inhibiting differentiation.

## Discussion

NFATc1 is induced by RANKL and the master regulator of osteoclastogenesis. On the other hand, transcription factors such as IRF8^(^ ^20^ ^)^ and MafB^(^ ^21^ ^)^ inhibit NFATc1 synthesis by mechanisms poorly understood.

We provide evidence that increased ThPOK, which occurs in malignancies, impairs osteoclast differentiation by regulating NFATc1 expression and function. Although ThPOK is essential for CD4^+^ T‐cell commitment, it is also present in non‐lymphoid tissues,^(22^ ^)^ and we find it in BMM osteoclast precursors. However, ThPOK expression by these cells decreases immediately after RANKL exposure, suggesting ThPOK might regulate formation of the bone resorptive polykaryon. We found that depletion of the transcription factor does not enhance the cytokine's capacity to promote differentiation of osteoclast precursors, which is in keeping with the diminished ThPOK expression after exposure to RANKL.

Because it is enhanced in various cancers, including lymphomas, we asked if, in contrast to lack of impact of its depletion, enhanced ThPOK influences osteoclast formation, which, in fact, it virtually eliminates. POK family proteins are characterized by two conserved motifs, namely a zinc finger DNA binding domain and a POZ/BTB region, which interacts with other transcription factors. The zinc finger region of LRF recognizes the NFATc1 promoter, thereby regulating its activity.^(^ ^15^ ^)^ Because ThPOK and LRF belong to the same subgroup of POK factors, on the basis of amino acid and nucleotide homology, we asked if excess ThPOK suppresses osteoclastogenesis by regulating NFATc1 transcription via binding its promoter. We find that ThPOK inhibits the cell's formation via Arg 389, within its second zinc finger domain. In contrast, inactive ThPOK^R389G^ fails to affect osteoclast formation and NFATc1 expression. ThPOK functions predominantly as transcriptional repressor in the control of cell lineage commitment since it was originally found blunting collagen promoter activity.^(^ ^23^ ^)^ Our data are consistent with this conclusion as they indicate ThPOK represses osteoclast differentiation by inhibiting NFATc1 transcription.

NFATc1 overexpression does not prevent the osteoclast‐inhibiting effects of excess ThPOK likely because they form a restrictive complex. Hence, like LRF, ThPOK blocks osteoclastogenesis by transcriptionally repressing NFATc1 in the early phase of osteoclast differentiation. LRF, however, is a NFATc1 transcriptional coactivator in mature osteoclasts. Although ThPOK also prevents NFATc1‐induced BMM differentiation into osteoclasts, unlike LRF, it does so in a non‐reversible manner. Hence, abundantly expressed NFATc1 fails to modify the inhibitory effect of ThPOK. Alternatively, ThPOK's reduction with RANKL administration and its impairment, when in excess of RANKL‐stimulated osteoclastogenesis, suggest that ThPOK diminution may be a necessary component of RANKL‐induced osteoclastogenesis.

Osteoclasts and immune cells share many molecules that regulate their differentiation and function. RANKL and NFATc1 were first identified in T lymphocytes, whereas RANK was originally noted in dendritic cells. Furthermore, Zap70, which is essential for T‐cell activation, negatively regulates osteoclast function.^(^ ^23^ ^)^ Our data establish ThPOK is a member of this T‐cell/osteoclast‐regulating family but exerts its effects on the bone‐resorptive cell only when in excess.

## Disclosures

All authors state that they have no conflicts of interest.

### Peer Review

The peer review history for this article is available at https://publons.com/publon/10.1002/jbm4.10613.
